# Attenuation correction synthesis for hybrid PET-MR scanners: validation for brain study applications

**DOI:** 10.1186/2197-7364-1-S1-A52

**Published:** 2014-07-29

**Authors:** Ninon Burgos, M Jorge Cardoso, Kris Thielemans, John S Duncan, David Atkinson, Simon R Arridge, Brian F Hutton, Sébastien Ourselin

**Affiliations:** Centre for Medical Image Computing, University College London, London, UK; Dementia Research Centre, University College London, London, UK; Institute of Nuclear Medicine, University College London, London, UK; Department of Clinical and Experimental Epilepsy, UCL IoN, London, UK; Centre for Medical Imaging, University College London, London, UK

In this work, we further validate a CT and attenuation map (μ-map) synthesis algorithm [1].

The CT synthesis method relies on a pre-acquired set of aligned MRI/CT pairs from multiple subjects. Each MRI from the database is non-rigidly registered to the target MRI. The CTs in the database are then mapped using the same transformation to the target MRI. A local image similarity measure between the target MRI and the set of registered MRIs is used as a surrogate of the underlying morphological similarity. Finally, the synthetic CT is generated using a voxel-wise weighting scheme, and converted to linear attenuation coefficients by a piecewise linear transformation.

Following the proposed method, a pseudo CT (pCT) was generated using only the MRI of the subject and compared to the ground truth CT, validating the accuracy of the CT synthesis. A PET image (PET_pCT_) was then reconstructed with an off-line version of the Siemens Healthcare reconstruction software using the pCT μ-map, and compared with the gold standard PET reconstructed using the CT μ-map.

**Figure 1:**
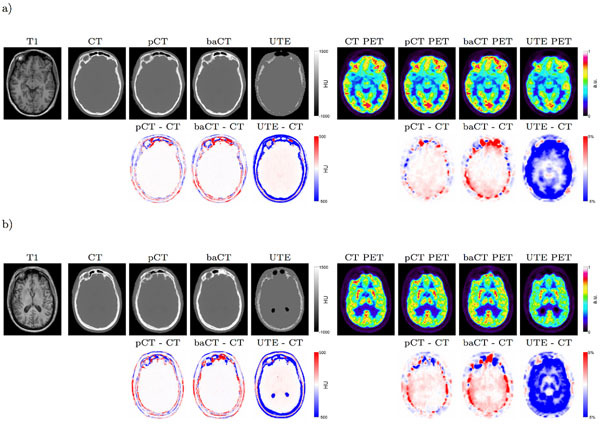
From left to right: The acquired T1-weighted MRI, the CT, pCT generated by the proposed, baCT and UTE CT, the gold standard FDG PET, pCT PET generated by the proposed method, baCT PET, and UTE PET (top row), and the difference images (bottom row) for the best (a) and the worst (b) subjects.

We validated our method for brain-related applications with 16 subjects and compared our solution to: a simpler atlas-based method, named the best-atlas method, obtained using a global similarity measure to select, from the database, the most similar template; and to the prototype version of a UTE-based method currently implemented on the first software versions of the Siemens Biograph mMR hybrid PET/MR scanners. The results presented in Table 1 demonstrate that the mean residual estimated between the PET_pCT_ and the gold standard PET is significantly smaller compared to the other methods. More accurate results are reached with the proposed method compared to the best-atlas method, which demonstrates the advantages of synthesising CTs at a local scale instead of a global scale (Figure 1).

**Table 1:** Average and SD of the mean absolute residual  and mean residual  between the ground truth CT and both the pseudo CT, best-atlas CT (baCT) and UTE CT (left column); average and SD of the relative MAR and relative MR between the gold standard CT PET and both the pseudo CT, best-atlas CT and UTE PETs (right column).

		CT (HU) - Head			PET (%) - Brain		
		pCT	baCT	UTE	pCT	baCT	UTE
MAR	Average	107	128	218	2.35	3.03	12.72
	SD	11.8	13.3	23.2	0.71	0.49	1.55
MR	Average	-7.2	18.0	-143	0.70	0.88	-12.61
	SD	14.9	16.3	35.0	1.32	1.45	1.60
